# The Effect of Metabolic Persian Diet on Ovulation Induction in Infertile Women

**DOI:** 10.1155/2023/6656779

**Published:** 2023-08-07

**Authors:** Nasrin Jamebozorg, Farzaneh Ghaffari, Fatemeh Alijaniha, Yasin Karimi, Robabeh Mohammad Beigi, Hamid Haghani, Mohsen Naseri, Leila Neisani Samani

**Affiliations:** ^1^Department of Midwifery, School of Nursing and Midwifery, Iran University of Medical Sciences, Tehran, Iran; ^2^School of Traditional Medicine, Shahid Beheshti University of Medical Sciences, Tehran, Iran; ^3^Traditional Medicine Clinical Trial Research Center, Shahed University, Tehran, Iran; ^4^School of Persian Medicine, Shahed University, Tehran, Iran; ^5^Department of Biostatistics, Faculty of Management and Information Technology, Iran University of Medical Sciences, Tehran, Iran; ^6^Department of Bio-Statistics, School of Health, Iran University of Medical Sciences, Tehran, Iran; ^7^Hikmat, Islamic and Traditional Medicine Department, The Academy of Medical Sciences, Tehran, Iran; ^8^Department of Medical Education, School of Medicine & Research Center of Medical Sciences Education, Iran University of Medical Sciences, Tehran, Iran

## Abstract

**Introduction:**

Infertility is an important issue with a high social and psychological burden. From the perspective of Persian Medicine (PM), uterine cold temperament is one of the important causes of female infertility.

**Aim:**

The aim of this study was to study the effect of PM or Iranian traditional medicine on improving ovulation and fertility.

**Methods:**

From January 2017 to August 2017, sixty infertile women with eugonadotropic ovulation disorder were randomly divided into two groups. Both groups were treated with clomiphene citrate, except that the intervention group also received metabolic Persian diet (MPD). In each cycle, the dominant follicles and endometrium were investigated with ultrasound. At the end of the cycle, beta human chorionic gonadotropin (*β-*HCG) was evaluated and if positive, another ultrasound was performed two weeks later to confirm early pregnancy.

**Results:**

The number of dominant follicles from the first to third cycle increased from 2.15 ± 1.14 to 2.28 ± 0.61 in the intervention group and decreased from 1.86 ± 0.79 to 1.30 ± 0.47 in the control group. The dominant follicle size changed from 16.00 ± 4.08 to 18.78 ± 2.78 and from 15.05 ± 3.11 to 15.80 ± 3.89 in the intervention and control groups, respectively. But endometrial thickness did not change significantly in the intervention group. Pregnancy results based on *β-*HCG and ultrasound findings were positive in 19 and 16 patients in the intervention group and in 10 and 7 patients in the control group, implying significantly higher numbers in the first group.

**Conclusion:**

It seems that MPD may be effective in the success of ovulation induction.

## 1. Introduction

Infertility, as one of the relatively common and serious problems among couples, is characterized by the absence of pregnancy after 12 months of unprotected intercourse [1, 2]. According to several studies, this problem is prevalent among about 15% of couples worldwide [3, 4]. Ovulatory disorders are one of the most common causes of infertility in women, and polycystic ovary syndrome (PCOS) has the largest share of these disorders [5]. The results of a study in Iran revealed that these disorders were involved in 57.7% of infertility among women, while PCOS alone in about 66% of cases was the underlying cause of ovulation problems [6].

Higher rates of psychiatric disorders and endometrial cancers in infertile women along with an increased risk of cardiovascular disease and metabolic disorders in women with PCOS, as reported in several studies, should be given more attention. In addition to these problems, the high cost of treatment is a major concern for infertile couples and a considerable burden on health system [7].

According to the World Health Organization, ovulatory disorders are divided into three categories. In the normogonadotrophic group (or group II disorders) accounting for about 85% of patients, PCOS is the major underlying cause. However, in this category, obesity and hormonal disorders, such as hyperprolactinemia or hypothyroidism, also cause infertility in a smaller percentage of women [8]. Hence, ovulation induction is the logical treatment for infertility of these patients. In addition to good effectiveness and better safety, it is a noninvasive and cheaper method than the *in vitro* method or IVF [9–11]. Nevertheless, despite the benefits of ovulation induction, according to several studies, the success rate of this method in fertilizing women is less than 20% [12]. In addition, IVF, despite the high costs, results in pregnancy and live birth in 37.3% and 29.6%, respectively [13, 14].

On the other hand, today, the importance of nutrition in infertility is somewhat known [15]. The association between preconception diet and the chance of pregnancy in women undergoing IVF/ICSI treatment was reported in a study. The results showed that improving nutritional status, according to Dutch dietary recommendation, increased fertility outcomes in patients [16]. Two other studies found the diet modifications as an effective factor and suggested that more adherences to the Mediterranean diet may help increase the chances of conceiving better outcomes for women undergoing IVF treatment [17]. In addition, numerous studies have examined the effect of diet on ovulatory disorders. One example would be the prospective study of more than 17,000 women over eight years, which found that high adherence to “fertility diet” (comprising increased intake of unsaturated fats and plant proteins) reduces the risk of infertility associated with ovulatory disorders in the future [18]. Also, a recent randomized clinical trial showed that a diet and lifestyle modification based on Persian medicine (PM) significantly increased the success of treatment in the women undergoing assisted reproduction [19].

PM is a valuable collection with long history of experience of Iranian physicians, being used in the field of prevention and treatment of various diseases [20]. Avicenna and Rhazes are among the most PM scholars influencing medical science for centuries, not only in Iran and Middle Eastern countries but also in Europe and the West [19, 21]. Increasing the quality of life in healthy people (21) and reducing the cost of diseases [22] are the major priorities in PM. Some interesting results of the studies inspired by the opinion of ancient scholars indicate that PM in the field of gynecology also has the ability to provide effective treatments [23, 24].

In general, from the perspective of PM, the first and most important step in the treatment of diseases is lifestyle modification, in which nutrition is of particular importance [25, 26]. According to PM scholars' point of view, foods are divided into two categories of hot and cold, while each of them affects the physiology of various body organs based on its temperament. Recent research has also confirmed the idea that the hormonal, autonomic, immune, and digestive systems each respond differently to hot and cold foods [25]. Moreover, according to PM, people are divided into two general groups of hot- and cold-tempered, whose immune and endocrine systems are significantly different and their diet should be adjusted based on their temperaments [27–29]. In modern medicine, there is a similar view called “personal medicine” and it pays attention to the wide individual differences in the prevention and treatment of diseases [30].

So, considering the temperamental view of PM about diseases and the fact that most types of infertility fall into the category of cold diseases, the first step of treatment would be to utilize a therapeutic diet that inclines the body and ovary temperament to heat [31]. To achieve this goal, metabolic Persian diet (MPD) is designed according to the constitutional perspective of PM, for this category of diseases that temporarily eliminates cold-tempered foods from the consumption of patients. In this diet, despite the elimination of cold-tempered foods, the view of modern knowledge of nutrition has also been completely considered, by providing all categories of food pyramid [32].

Given the importance of infertility and high costs, an attempt was made to investigate the effect of MPD as a simple, inexpensive, and uncomplicated method on the success of ovulation induction.

## 2. Materials and Methods

The current study was a quasi-experimental clinical trial (intervention and control group) performed on patients referred to an academic infertility center of Akbarabadi Hospital in Tehran, Iran, from January 2017 to August 2017. Herein, the objectives of the study, being in full compliance with the Helsinki Declaration, were explained to the patients and their written consent was obtained.

### 2.1. Participants

Eligible participants in this study were women aged 20–35 years with ovulation-related infertility, who have experienced less than three courses of ovulation induction.

#### 2.1.1. Inclusion Criteria

Those who volunteered to participate in the study and filled out the consent form, who do not have any chronic physical disease, who do not have history of mental health problems, and have ovarian-associated infertility and serum FSH (follicle-stimulating hormone) levels between 1 and 15 IU/L were included in the study.

#### 2.1.2. Exclusion Criteria

Participants who interrupt the diet for more than 24 consecutive hours or do not perform ultrasound and paraclinical evaluations will be excluded from the study.

### 2.2. Study Design

In the current work, the continuous sampling method was performed on two different days. On the first day of attending the infertility center, through the lottery method, it was determined that the samples were included in the intervention group and the next day in the control group until the end of the sampling stage; hence, preventing the control group from receiving the diet.

To determine the required sample size at 95% and a power of 80%, and to evaluate the effect of MPD on the follicle growth in women undergoing ovulation induction, if the effect of dietary intervention on the follicle growth compared to the control group is equal to 2.5 (*d* = 2.5) to make this intervention statistically significant, after quantification in the following formula, the required sample volume was estimated to be 60.

(1) $$\begin{array}{c} n=\left(Z1-\alpha +Z1-\beta \right)\;2\times \frac{\left(S12+S22\right)}{d2}\text{,}\\ d=\frac{2}{5}\text{,}\\ S1=\frac{4}{5}\text{,}\\ S2=1\text{,}\\ Z1-\alpha =\frac{1}{96}\text{,}\\ Z1-\beta =\frac{0}{84}\text{.} \end{array}$$

Sample size in each group was estimated to be *N* = 23. Assuming a sample drop of 10%, the required sample volume was estimated to be *N* = 30. In addition, based on part of our pilot study conducted on 12 patients, the standard deviation of follicle growth in the control and intervention groups was estimated to be 4.5 and 1, respectively.

The information of participants was obtained through interviews and the data in medical records. A researcher-made demographic questionnaire included 25 questions about the couple's age, education level, and occupation along with the patient's fertility characteristics, including the number of pregnancies, deliveries, and abortions, history of infertility, method and the duration of contraception, menstrual characteristics, the rate of sexual intercourse, and anthropometric indices. This information was obtained in order to investigate the real effect of the intervention on fertility via matching study groups.

In addition to the initial nutritional assessment at the beginning of the study using the 24-hour dietary recall questionnaire, to check adherence with diet, this questionnaire was completed twice more by patients: in the middle of the study (six weeks following the intervention) and at the end of the third cycle. However, for women who became pregnant before six or 12 weeks, 24-hour dietary recall was not checked in the second and third rounds, respectively. It should be noted that 80% adherence to the diet was accepted. The data related to the 24-hour dietary recall questionnaire were investigated by a nutritionist with food processor software and *N*4, and the information about the diet plan was obtained.

### 2.3. Intervention

In the present study, all patients were evaluated for three menstrual cycles. In the first cycle, in order to induce the ovulation, patients of both groups took 100 mg of clomiphene citrate daily from day three to seven of menstruation. If pregnancy did not occur in each cycle, the dose of clomiphene citrate was repeated in the second and third cycles.

The necessary instructions in the MPD were explained to the patients in the intervention group. The training was provided in the form of face-to-face and pamphlet training. Patients had to follow this diet as soon as the treatment with clomiphene citrate started in the first cycle. Patients in the intervention group had to follow this diet until the end of the third cycle or until pregnancy was confirmed before the end of the third cycle. In contrast, the control group did not receive any instructions and were monitored for three cycles. The diet plan provided to patients is described in Table 1.

### 2.4. Outcome Measure

The first main outcome of the study was related to ovarian and uterine findings and the second one was related to fertility. The first one included the number of dominant follicle, dominant follicle size, and endometrial diameter. These parameters were assessed via ultrasonography ultrasound on day 14 of the menstrual cycle. If the dominant follicle size was between 18 and 20 mm in ultrasonography, injectable human chorionic gonadotropin (HCG) at a dose of 5,000 units was prescribed by an infertility fellowship. The ultrasonography of all patients was performed by the same person in one place (Hounds electric HS2100 ultrasonography device, made in Japan, 2013).

Fertility outcome was performed in two ways: laboratory test and ultrasonographic examination. In this method, the *β-*HCG test was performed between days 28 and 30 of menstruation. To measure serum *β-*HCG, an enzyme-linked immunosorbent assay (ELISA) reader with awareness stat fax 3200 model was employed. In *β-*HCG-positive individuals, ultrasonography was performed again at weeks six to seventh, the results, including the type of pregnancy in terms of implantation location and fetal heart rate, were recorded in the data sheet. On the other hand, in *β-*HCG-negative patients the mentioned diagnostic protocol was repeated in the second and third cycles.

### 2.5. Statistical Analysis

Data were analyzed applying Statistical Package for Social Sciences (SPSS) software version 21. Significance level was considered less than 0.05. After entering the information into the software, the data were categorized on a regular basis. The normality of the variables was assessed with the test. Descriptive statistics were utilized to adjust the frequency distribution table and calculate the central indicators (mean and standard deviation) whose output was in the form of a table. In addition, in order to achieve the goals and answer the research questions, paired *t*-test and independent *t*-test were employed. The analysis of data on the education level of participants and their spouses was performed by Chi-square test and Fisher's exact test.

## 3. Result

### 3.1. Patients

**Figure 1:**
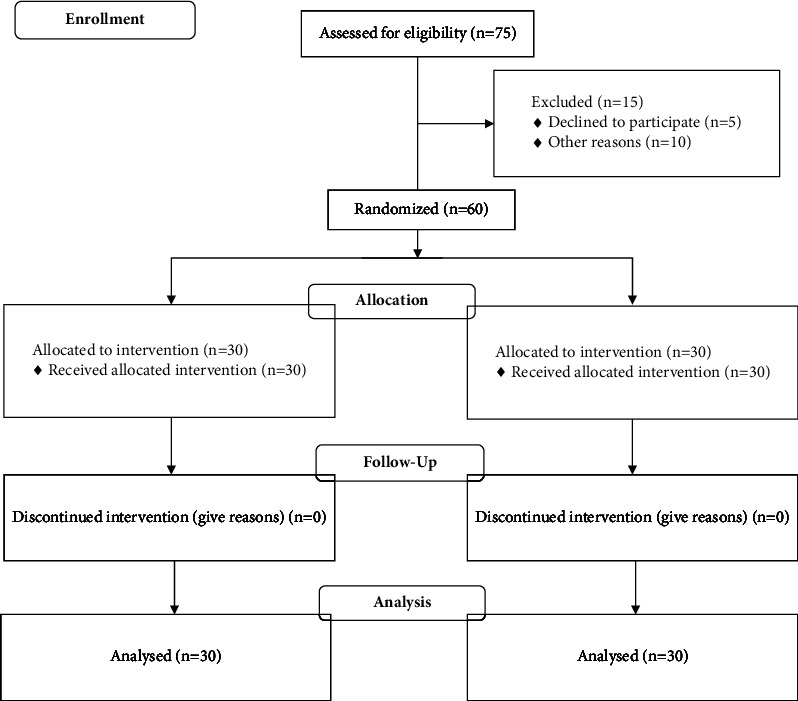
Consort diagram. Flow diagram graphically describes the design of the study: enrolment, intervention, follow-up, and data analysis.

All sixty patients completed the study and second and third round evaluations with 24-hour food recall questionnaire showed that adherence to MPD for all patients in the intervention group was within the desired level above 80%. The expected follow-up was also performed by all patients (Figure 1).

The demographic characteristics of the study groups are represented in Table 2. There was no significant difference between the two groups in terms of age of husband, BMI, number of marriages, duration of marriage, education level of patient and husband, employment status of patient and husband, and their economic status.

Moreover, the surveys on the fertility characteristics of the studied women indicated that there was no significant difference between the two groups. These characteristics are expressed in Table 3.

### 3.2. Main Findings

As shown in Table 4, considering the size and number of dominant follicles in the first and second cycles, there was no significant difference between the study groups after the start of the intervention; yet, in the third cycle, the ultrasonographic parameters in women receiving the MPD were significantly higher than the control group (*P* value <0.001 and *P* value = 0.019). However, the results obtained from the endometrial diameter did not reveal any significant differences in any of the three cycles following the intervention.

Nevertheless, regarding the most important parameters of this study, namely, pregnancy, laboratory results of *β-*HCG and ultrasonography implied that in the intervention group, the rate of pregnancy was significantly (30%) higher than that in the control group.

## 4. Discussion

In the current study, in order to investigate the real effect of MPD on the success rate of ovulation induction, study groups were matched in terms of demographic, social, and reproductive parameters. The results implied that MPD is significantly effective in fertility in women with ovulation disorders. This diet has increased the chances of pregnancy in infertile women by 30%. These results prove the opinion of Persian physicians declaring that the constitutionally warm diet has a positive effect on cold-tempered diseases such, as female infertility.

Our previous study showed the effectiveness of a special diet in improving the symptoms of functional dyspepsia. Due to PM perspective considering indigestion as a chronic disease caused by cold and wet temperament of the stomach, foods that require a lot of energy for digestion and are considered extremely cold, wet, heavy, and harmful to the stomach in the mentioned viewpoint were excluded from patients' diet. The results showed that in the group receiving the metabolic Persian diet (MPD), indigestion, loss of appetite, epigastric night pain, and epigastric irritation were significantly improved compared to the beginning of the trial. The reduction of a wide range of symptoms following this diet is a significant finding because common treatments such as PPI and prokinetics only improve some of the symptoms [33].

Also, another recent clinical study showed the effectiveness of a traditional medicine-based lifestyle and diet on infertility treatment in women undergoing assisted reproduction. The diet that was used in the mentioned study was also designed based on warm-tempered foods and adjusted according to the temperament of each patient [34].

Indeed, other studies have addressed the importance of nutrition and diet in female infertility. For instance, in the study of Twigt et al., the effect of nutrition on the success of IVF/ICSI treatment has also been highlighted. Their results indicated that for each increase in Preconception Dietary Risk (PDR) score, an indicator for measuring the quality of nutrition, the incidence of pregnancy with assisted reproductive techniques was increased by 65% [16].

A prospective study of more than 18,000 women of childbearing age also found that women consuming the most low-fat dairy had a 1.85-fold higher chance of an ovulatory infertility than women consuming the least [35].

In a recent trial, like this study, examining a MPD along with teaching healthy eating habits, the results showed pregnancy rate after 3 months in the intervention and control groups were 35.2% and 12.4%, respectively, in infertile women under IVF. Also, the number of ovum number and mature ovum number increased significantly under the influence of these trainings. However, unlike the present study, it does not offer a therapeutic diet for the cold temperament, but examines a healthy diet that recommends relatively hot- or cold-tempered foods that produce healthy humors in the body [34].

In PM, to manage the disease, food should be evaluated from two aspects: quantity and quality. In terms of quantity, fasting and calorie restriction are among the therapeutic strategies applied in certain conditions [16]. However, once the temperament of the body or an organ, such as the ovary is out of balance and tends to hotness or coldness, the quality of the foods is also taken into consideration. In this case, the use of foods or in the second step, drugs with the opposite temperament can restore the body's homeostasis.

So far, few studies have examined the concept of temperament, one of these which showed some differences in neuroendocrine in people with different temperaments. The sympathetic nervous system is significantly more active in hot-tempered people, whereas the parasympathetic nervous system, sympathoadrenal system, and corticosteroid secretion are more active in cold-tempered people [27].

An animal study revealed that hot-tempered food significantly increased thyroid hormones and urine vanillylmandelic acid (VMA). On the contrary, adding cold-tempered seeds to the rats' diet increased serum cortisol and decreased the VMA levels. Changes in urinary VMA levels indicated an increased activity of the sympathetic nervous system following the consumption of hot-tempered grains and vice versa [36].

The effect of hot foods on thyroid function is when this hormone plays an important role in female fertility. According to several reports, up to 11.3% of infertility-related disorders present with subclinical hypothyroidism [8]. Sex hormones are no exception to this rule and are affected by food and diet. Several studies have linked the increased dairy consumption to the change in female sex hormones. One study performed on 259 women with regular menstruation showed that the increased dairy consumption was associated with decreased serum estradiol. It was also revealed that consuming cream and yogurt raises the risk of sporadic anovulation [37]. This is while in MPD, most dairy foods with a cold temperament from the perspective of PM, such as yogurt, yogurt drink, and cream, are temporarily banned. However, the mechanism of the positive effect of MPD on the success of ovulation induction is not exactly clear. This requires further studies in the future. It is also recommended that the effect of this diet on other causes of infertility, including male and female causes be evaluated. Moreover, studying the effect of MPD on IVF success can be helpful in demonstrating the effect of this lifestyle-related treatment on infertility. Using modern analytical techniques to determine the chemical characteristics of the MPD can be the subject of further studies to standardize this diet.

## 5. Conclusion

The results of this study indicated that the use of MPD (by eliminating cold-tempered foods) may improve the success of ovulation induction in infertile women. It seems that this simple and uncomplicated diet could be considered to be a part of infertility treatment.

## Figures and Tables

**Table 1:** Summarized MPD plan.

Main food group (unit)	Sample	
	Banned food	Preferred food
Bread and cereals (6 or more)	Unfermented bread pasta, lasagna, and pizza	Well-fermented and toasted bread and cooked rice by removing the glaze
Fruits and vegetables (4 or more)	All cold fruits (watermelon, peach, nectarine, kiwi, strawberry, orange, tangerine, sweet lemon, and sour fruits) are prohibited. Mushrooms and potatoes except when boiled are banned	Apples, grapes, olives, dates, figs, sweet pomegranates, cooked vegetables, carrot juice, apple juice, and raisins
Meat and eggs (2 or more)	Fish other than salmon when steamed or cooked beef and veal meat kielbasa, burgers, tuna fish, and canned food	Chicken and poultry meat semifried egg yolk and lamb meat
Milk and dairy (2 or more)	Yogurt drink, yogurt, ice cream, curd, old cheese, cream, and buttermilk	Milk and a little candy (nabāt) or honey and pasteurized cheese
Nuts, seeds, and beans	Walnuts, almonds, hazelnuts, pistachios sunflower seeds, and the like	Peas, beans, lentils, beans, chickpeas, and mung beans^*∗*^
Other	Solid and liquid oils (except olive and sesame oil), tallow, tail, butter, cream, buttermilk and especially fatty sauces sweets, especially candy and chocolate, spices, peppers, cinnamon, pickles and salty foods, coffee, strong tea, soft drinks, and industrial juices	Olive or sesame oil honey, sugar, homemade jam, salt and turmeric in small amounts, and light tea in small amounts and not immediately after meals

^*∗*^Preparation method: soak the beans for up to 2 hours, remove the remained water, and then cook by adding a little salt or preferably with half the equivalent amount of rice.

**Table 2:** Demographic characteristics.

Characteristic	Different subgroups	Intervention (*N* = 30)	Control (*N* = 30)	*P* value
Age	—	32.53 ± 2.40	32.27 ± 2.35	0.665

Husband's age	—	37.67 ± 4.49	37.37 ± 5.36	0.815

BMI	—	23.9 ± 1.48	24.56 ± 1.88	0.139

Number of marriages	—	1.10 ± 0.3	1.10 ± 0.3	<0.999

Duration of marriage	4–6 years	9 (30%)	8 (26.7%)	0.133
	7–9 years	7 (23.4%)	14 (46.6%)	
	≥10 years	14 (46.6%)	8 (26.7%)	

Education level	Middle school	3 (10%)	4 (13.3%)	0.333
	High school	6 (20%)	11 (36.7%)	
	University	20 (66.7%)	15 (50%)	
	Religious education	1 (3.3%)	0 (0%)	

Husband's education level	Middle school	3 (10%)	4 (13.3%)	0.746
	High school	7 (23.3%)	9 (30%)	
	University	20 (66.7%)	17 (56.7%)	

Occupational status	Employed	12 (40%)	17 (56.7%)	0.196
	Housewife	18 (60%)	13 (43.3%)	

Husband's occupational status	Unemployed	2 (6.7%)	1 (3.3%)	0.761
	Self-employed	8 (26.6%)	6 (20%)	
	Employee	13 (43.3%)	17 (56.7%)	
	Worker	7 (23.3%)	6 (20%)	

Economic situation	Undesirable	3 (10%)	0 (0%)	0.345
	Relatively desirable	19 (63.3%)	22 (73.3%)	
	Desirable	8 (26.7%)	8 (26.7%)	

**Table 3:** Fertility characteristics (continued).

Characteristic	Different subgroups	Intervention (*N* = 30)	Control (*N* = 30)	*P* value
Number of pregnancies	—	1.03 ± 1.00	1.23 ± 1.13	0.472

Number of deliveries	—	0.23 ± 0.43	0.200 ± 0.41	0.759

Number of children	—	0.13 ± 0.34	0.10 ± 0.30	0.649

Number of marriages	—	1.10 ± 0.3	1.10 ± 0.3	<0.999

Number of abortions	—	0.73 ± 0.83	1.03 ± 0.90	0.182

Type of abortions	Miscarriage (spontaneous abortion)	11 (73.3%)	12 (63.2%)	0.715
	Induction abortion	4 (26.7%)	7 (36.8%)	

Curettage	—	4 (13.3%)	7 (23.3%)	0.317

Type of previous deliveries	Vaginal delivery	3 (10%)	3 (10%)	0.774
	Cesarean section	4 (13.3%)	3 (10%)	
	No previous delivery	23 (76.7%)	24 (80%)	

Type of fertility	Primary	12 (40%)	14 (48.3%)	0.522
	Secondary	18 (60%)	15 (51.7%)	

Method of contraception	Discontinued contraception	16 (53.4%)	17 (56.7%)	0.220
	Condom	10 (33.3%)	5 (16.6%)	
	Combined pill	4 (13.3%)	8 (26.7%)	

Age of first menstruation (menarche)	—	12.87 ± 1.45	12.77 ± 1.41	0.788

Menstrual pattern	Regular	13 (43.3%)	13 (43.3%)	<0.999
	Irregular	17 (56.7%)	17 (56.7%)	

Duration of menstruation	—	6.23 ± 0.97	6.20 ± 1.09	0.901

Menstrual interval	≤28	13 (43.3%)	12 (80%)	0.573
	29–30	12 (40%)	12 (40%)	
	≥31	5 (16.7%)	6 (20%)	

Menstrual bleeding volume	High	7 (23.3%)	8 (26.7%)	0.953
	Medium	17 (56.7%)	16 (53.3%)	
	Low	6 (20%)	6 (20%)	

Menstrual pain	Yes	15 (50%)	13 (43.3%)	0.605
	No	15 (50%)	17 (56.7%)	

Number of sexual intercourses per week	—	2.30 ± 1.18	2.30 ± 1.18	<0.999

**Table 4:** Comparison of sonographic and laboratory results of the two groups.

Characteristic	Different subgroups	Intervention (*N* = 30)	Control (*N* = 30)	*P* value
Number of dominant follicles	1^st^ cycle	2.15 ± 1.14	1.86 ± 0.79	0.471
	2^nd^ cycle	1.80 ± 0.89	1.65 ± 0.71	0.681
	3^rd^ cycle	2.28 ± 0.61	1.30 ± 0.47	<0.001

Size dominant follicle	1^st^ cycle	16.00 ± 4.08	15.05 ± 3.11	0.404
	2^nd^ cycle	15.20 ± 3.39	16.65 ± 2.79	0.131
	3^rd^ cycle	18.78 ± 2.78	15.80 ± 3.89	0.019

Endometrial diameter	1^st^ cycle	6.37 ± 1.19	6.50 ± 1.11	0.655
	2^nd^ cycle	6.73 ± 1.08	6.70 ± 0.83	0.894
	3^rd^ cycle	7.04 ± 1.26	7.53 ± 1.16	0.150

*β*-HCG test	Negative	11 (36.7%)	20 (66.7%)	0.020
	Positive	19 (63.3%)	10 (33.3%)	

Pregnancy based on ultrasound	Negative	14 (46.7%)	23 (76.7%)	0.017
	Positive	16 (53.3%)	7 (23.3%)	

## Data Availability

The data that support the findings of this study are available upon request from the corresponding author, L. Neisani Samani.
